# Visual-Inertial-Wheel Odometry with Slip Compensation and Dynamic Feature Elimination

**DOI:** 10.3390/s25051537

**Published:** 2025-03-01

**Authors:** Niraj Reginald, Omar Al-Buraiki, Thanacha Choopojcharoen, Baris Fidan, Ehsan Hashemi

**Affiliations:** 1 Department of Mechanical and Mechatronics Engineering, University of Waterloo, 200 University Ave W, Waterloo, ON N2L 3G1, Canada; nnreginald@uwaterloo.ca (N.R.); omar.alburaiki@uwaterloo.ca (O.A.-B.); tchoopoj@uwaterloo.ca (T.C.); 2 Mechanical Engineering Department, University of Alberta, Edmonton, AB T6G 2R3, Canada; ehashemi@ualberta.ca

**Keywords:** wheel slippage compensation, multi state constraint Kalman filtering, multi-sensor fusion, visual-inertial-wheel odometry, dynamic environment navigation

## Abstract

Inertial navigation systems augmented with visual and wheel odometry measurements have emerged as a robust solution to address uncertainties in robot localization and odometry. This paper introduces a novel data-driven approach to compensate for wheel slippage in visual-inertial-wheel odometry (VIWO). The proposed method leverages Gaussian process regression (GPR) with deep kernel design and long short-term memory (LSTM) layers to model and mitigate slippage-induced errors effectively. Furthermore, a feature confidence estimator is incorporated to address the impact of dynamic feature points on visual measurements, ensuring reliable data integration. By refining these measurements, the system utilizes a multi-state constraint Kalman filter (MSCKF) to achieve accurate state estimation and enhanced navigation performance. The effectiveness of the proposed approach is demonstrated through extensive simulations and experimental validations using real-world datasets. The results highlight the ability of the method to handle challenging terrains and dynamic environments by compensating for wheel slippage and mitigating the influence of dynamic objects. Compared to conventional VIWO systems, the integration of GPR and LSTM layers significantly improves localization accuracy and robustness. This work paves the way for deploying VIWO systems in diverse and unpredictable environments, contributing to advancements in autonomous navigation and multi-sensor fusion technologies.

## 1. Introduction

In wheeled mobile robot navigation, visual inertial navigation system (VINS) algorithms are considered state of the art for localization in GPS-denied environments [1,2,3]. The initialization procedure of VINS algorithms significantly influence the overall accuracy and robustness of the algorithm [4,5]. The initialization methods can be categorized as joint versus disjoint ones [6]. Joint methods estimate both the camera trajectory and inertial parameters simultaneously within a tightly coupled framework. This approach ensures global consistency in the estimates, reducing error propagation. However, joint methods often require higher computational resources and are more complex to implement, making them less practical for real-time applications. On the other hand, the disjoint methods assume that an accurate camera trajectory can be derived through monocular vision, and the inertial parameters are then estimated by using this trajectory.

Many of these algorithms rely on the use of monocular vision due to its affordability. However, they present inherent challenges, particularly with degenerate motions that introduce unobservable directions in the estimation process [7]. These motions, often uniform linear or circular, make scale and orientation unobservable, compromising the system’s robustness and accuracy [8]. To address these issues, researchers have integrated wheel odometry (WO) measurements into VINS, resulting in robust initialization and improved performance [9,10,11]. The existing VIWO schemes mainly focus on fusing WO information in the backend while ignoring it in the front end [12] and often assume little to no wheel slippage, which does not hold in practice [13]. Furthermore, side-slip effects pose challenges for tracking cornering trajectories, which require additional adaptive control techniques, observer design, and trajectory planning schemes [14,15]. Hence, when augmenting a VINS with a WO one, the longitudinal and lateral errors due to wheel slippage need to be taken into account and compensated.

Many studies in the literature on slip compensation rely on accurate slip ratio models [16], which may not hold under varying surface conditions. As an alternative, Gaussian process (GP) modeling can encode prior distributions over functions, allowing updates to derive posterior distributions based on new test data. Leveraging this feature of GP modeling, regression tasks can be carried out where estimates of a function model are obtained given observed input–output data [17]. Notably, in the recent past, GP regression (GPR) has been used for learning the inverse dynamics of systems [18,19]. Unlike these model-based approaches, Liu et al. address slip effects by detecting and omitting updates during slip events using a threshold-based method, ensuring real-time performance but leaving the underlying slip dynamics unmodeled [20]. In contrast, our work utilizes GPR alongside other tools to estimate wheel slippage continuously, providing a more robust and adaptive compensation strategy.

In visual odometry (VO) estimations using sparse features, random sample consensus (RANSAC) is used as the preferred outlier handling mechanism [21]. However, RANSAC’s performance is inadequate when a significant portion of feature points are in motion or when dynamic objects obstruct the camera’s field of view (FOV) [22]. To address this issue, additional sensory data, such as those obtained from IMUs, are utilized to evaluate the reliability of matched features and eliminate dynamic feature points [23], which represent a preliminary version of the results of this article.

Recent state-of-the-art visual-inertial-wheel odometry (VIWO) algorithms have focused on addressing key challenges such as long-term drift, sensor calibration, and multi-modal data integration. The robust neural gyroscope calibration VIWO (RNGC-VIWO) system by Zhi et al. reduces gyroscope drift and improves localization accuracy through neural gyroscope calibration, while the approach by Lee et al. performs online calibration of wheel encoders’ intrinsic and extrinsic parameters to maintain consistent performance across various terrains [24]. Additionally, the partial invariant extended Kalman filter VIWO (PIEKF-VIWO) algorithm by Hua et al. enhances positioning accuracy by leveraging a partial invariant extended Kalman filter to optimally fuse wheel measurements with kinematic constraints [25]. Similarly, Zhou et al. propose the real-time appearance-based mapping VIWO (RTABMAP-VIWO) method, which uniquely incorporates wheel odometry and IMU data into RTABMAP using an extended Kalman filter (EKF) to reduce local cumulative errors in simultaneous localization and mapping (SLAM) systems [26]. However, despite these advancements, a common area for improvement remains in accurately compensating for wheel slippage, which can introduce significant errors in localization, particularly in challenging terrains or during dynamic maneuvers. Most existing methods do not fully account for the longitudinal and lateral slip effects, leading to reduced accuracy in real-world applications.

Two other effective technical tools used in the literature for improving VINS and VIWO results via modeling and mitigating slippage-induced errors are recurrent neural network (RNN) and long short-term memory (LSTM) [27]. These tools are particularly useful in learning sequential correlations of a sensor’s error propagation with time to more accurately position a vehicle. LSTM networks have also been employed in global navigation satellite system (GNSS)-free navigation systems to correct drift in velocity estimates derived from optical flow measurements [28]. In such drift correction, optical odometry and radar height estimates are fused with LSTM-based velocity corrections to mitigate the effects of GNSS signal loss. Similarly, deep-learning-based visual odometry (VO) methods, such as recurrent convolutional neural networks (RCNNs) and bi-directional LSTM (Bi-LSTM) architectures, have been used to improve pose estimation accuracy by capturing both spatial and temporal dependencies [29,30]. These methods, including frameworks like MagicVO, leverage CNNs for feature extraction and Bi-LSTM for sequential modeling, outperforming traditional VO systems in pose estimation accuracy and generalization ability.

A preliminary version of the results of this article has appeared in [23]. In contrast to [23], this work introduces a data-driven methodology employing GPR and LSTM networks to address wheel slippage, which is not previously addressed in [23]. This integration of GPR and LSTM enhances the prior approach substantially via effectively mitigating errors induced by slippage; thus, it significantly improves the precision of wheel odometry measurements. Furthermore, this work advances our earlier methodology in [23] by integrating a feature confidence estimator that utilizes additional sensory data. This ensures that only reliable sensor data influences the system’s state estimation. The advancements presented in this work are rigorously validated through extensive experimental trials using real-world datasets, demonstrating the methodologies’ capability to effectively manage challenging terrains and dynamic environments. In addition, a GPR [31] scheme is designed using a deep kernel approach with LSTM layers that are known to capture spatio-temporal variations [32]. The WO error residuals are learned between the physical prediction and the predicted ground truth data obtained using a differential drive kinematic model and the history of control input values. The refined WO measurements are then fused in a multi-state constraint Kalman filter (MSCKF) [33] framework for state estimation. Comparative simulations based on real-world data and indoor experiments reveal the performance improvement using the proposed approach.

In summary, this paper introduces a novel data-driven methodology to enhance visual-inertial-wheel odometry (VIWO) by addressing wheel slippage and improving localization accuracy. The key contributions of this work are

(i) The use of a data-driven methodology employing GPR and LSTM networks to address wheel slippage. This integration of GPR and LSTM presents a substantial enhancement over prior approach by effectively mitigating errors induced by slippage; thus, it significantly improves the precision of wheel odometry measurements.
(ii) The incorporation of a feature confidence estimator that utilizes additional sensory data. This ensures that only reliable sensor data influence the system’s state estimation.

A comprehensive comparative analysis of the key features of our work with respect to some related state-of-art works in the literature on VIWO/VINS and some recently developed deep-learning-based VO algorithms are presented in Table A1 and Table A2 in the Appendix A. The remainder of this article is structured as follows: Section 2 outlines the system description and preliminary details. Section 3 introduces the proposed method for compensating wheel slippage and other inherent errors associated with WO. The experimental results are presented and analyzed in Section 6. Finally, Section 7 concludes the paper and discusses future research directions.

## 2. The System Description and Preliminaries

**Figure 1:**
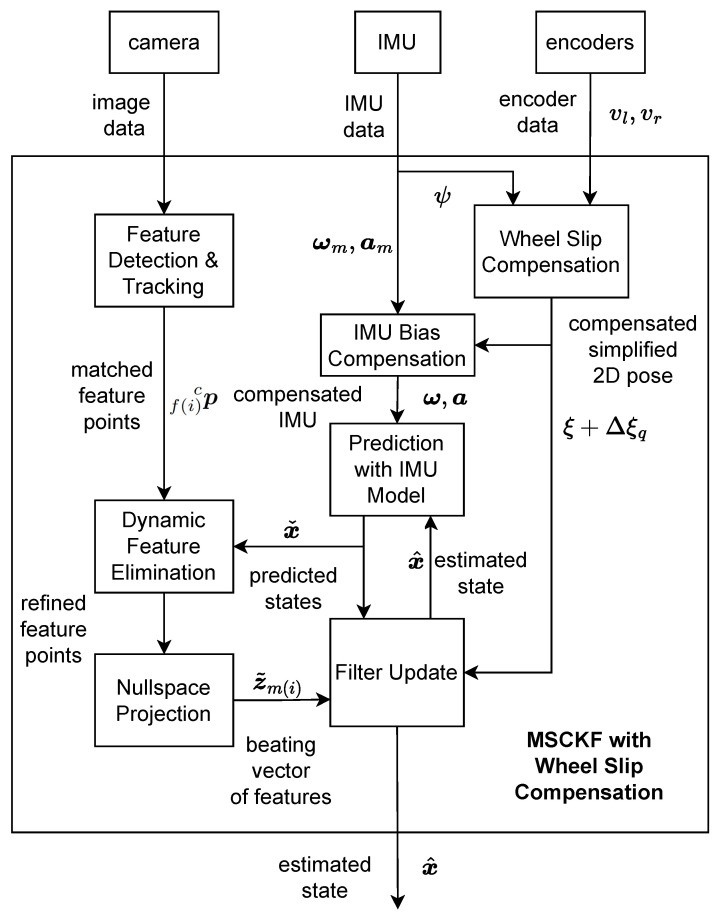
Overall structure of the proposed VIWO scheme.

In this section, we briefly describe the key components of the tightly coupled visual inertial wheel odometry (VIWO) system. To describe the system, we first define the frame notations. The notations (·)b, (·)w, (·)c, (·)o denote the body-fixed IMU {*b*}, world {*w*}, camera {*c*}, and WO {*o*} frames, respectively. The origin of frame {*o*} is at the center of the axle connecting the two wheels of the robot. Furthermore, R represents a rotation matrix, and Rok represents the rotation in {*o*} frame when the kth image is taken. The following sections describe the key measurements of the system. The system receives measurements from camera, IMU, and wheel encoders. Figure 1 represents the key overall system components of our proposed method.

### 2.1. IMU Kinematics Model

An IMU provides angular velocity $\omega_{m}$ and linear acceleration $a_{m}$, which can be measured as [34]

(1) $$\omega_{m}=\omega +b_{g}+n_{g},$$

(2) Sam=a+RGIg+ba+Sna,

where $\omega \in R^{3}$ and $a\in R^{3}$ are the true angular velocity and acceleration, g is a vector of gravitational acceleration, S is a symmetric matrix representing the scale factors, and $n_{g}$ and $n_{a}$ are the considered zero-mean Gaussian noises with $E\left[n_{g}\right]=0,E\left[n_{a}\right]=0$.

Both scale factors and biases can be determined and integrated into the IMU signal processing prior to their application in the multi state constraint Kalman (MSCKF) measurement update. The scale factor of the IMU is determined based on the methodology outlined in reference [5,35]. The IMU biases $b_{a}$ and $b_{g}$ are computed through a least squares optimization procedure. To determine the gyroscope bias, the optimization aims to minimize the difference between the rotation calculated from the pre-integrated IMU data and the one derived from WO [36]. Similarly, the accelerometer bias is estimated using the WO translation.

Once the biases are estimated or known, one can compensate for the bias and determine the translation of the robot with respect to the world frame pbw and the rotation q of the robot qwbk, which can be determined by solving the following differential kinematics equations [37]:

(3) ddt(qwb)=12Ω(ωm−bg)qwb

(4) ddt(pbw)=vbw

(5) ddt(vbw)=Sam−RGIg−ba

where Ω(·) is the skew-symmetric operator on a 3-dimensional vector. These three equations serve as references for the discrete-time process model of the proposed MSCKF.

### 2.2. Camera Measurement Model

The visual measurements corresponding to feature *i* measured by the camera projection function $\pi \left({\left[x,y,z\right]}^{⊤}\right)={\left[\frac{x}{z},\frac{y}{z}\right]}^{⊤}$, are given as

(6) zm(i)=π(pf(i)c)+ni,

(7) pf(i)c=RbcRwb(pf(i)w−pbw)+pbc,

where pf(i)w is the 3D feature position, $n_{i}$ is the measurement noise, Rbc and pbc are the camera to IMU extrinsic information. Equation (6) is primarily used as a reference for a measurement model on refined feature points after static feature point selection. As stated in [23], nullspace marginalization can be applied to (6) to obtain a new measurement equation that is independent of feature positions, resulting in a substantial reduction in computation and the need for storing feature positions in the state vector of MSCKF.

Filtered measurement estimates ${\hat{z}}_{m(i)}$ and the state estimate $\hat{x}$ are obtained by Kalman filtering based on the residuals ${\tilde{z}}_{m(i)}=z_{m(i)}-{\hat{z}}_{m(i)}$, $\tilde{x}=x-\hat{x}$ and the measurement model

(8) z˜m(i)=Hx,ix˜+Hf,ip˜f(i)w+ni

where $H_{x,i}$ is the Jacobian related to the robot state, and $H_{f,i}$ is the Jacobian related to feature position. Stacking the residuals ${\tilde{z}}_{m(i),k}$ for all feature points *i* and computing the nullspace marginalization as in (8), a camera measurement residual process model independent of the feature positions is obtained as

(9) $${\tilde{z}}_{m(i)}=H_{x}\tilde{x}+n_{i},$$

which is directly used in the multi state constraint Kalman filter (MSCKF) design to estimate the robot states, as in [11]. Since the feature positions are instantly marginalized from the state vector, this improves the computational efficiency of the filter, making it suitable for resource constrained platforms and applications.

## 3. Overall Methodology

The overall structure of the proposed multi-state constraint Kalman filter (MSCKF) is discussed in this section. Figure 1 shows the block diagram with key components of the proposed system. The MSCKF takes three sets of measurements: camera images, 6-axis IMU measurements, and wheel encoder measurements. Each set of raw data measurements is processed in an interconnected manner before being fed through the measurement update of the filter.

### 3.1. Robot State Vector

For any kth iteration of sampling, the computation or sampling is performed at a sampled time $t_{k}$. At each sampled time, the state vector $x_{k}={\left[\begin{array}{ccc} x_{I_{k}}^{⊤} & x_{C_{k}}^{⊤} & x_{O_{k}}^{⊤} \end{array}\right]}^{⊤}$ of the robot consists of the current inertial state,

(10) xIk=q¯wbk⊤pbkw⊤vbkw⊤bg⊤ba⊤⊤,

and the lumped vector of the past *n* world to inertial frame transformations

(11) xCk=q¯wbk−1⊤pbk−1w⊤⋯q¯wbk−n⊤pbk−nw⊤⊤,

where q¯wbj denotes the quaternion corresponding to the rotation Rwbj and a translation pbjw at time step *j*, for j=k−n,⋯,k. pbkw and vbkw are the position and velocity vectors, and $b_{g}$ and $b_{a}$ are the gyroscope and accelerometer biases, respectively. Furthermore, xOk=q¯okw⊤pokw⊤⊤ consists of the rotation and position information obtained using wheel encoder data.

### 3.2. Process Model

The proposed MSCKF uses the generic IMU nonlinear kinematics process model to propagate the state x by numerically integrating Equations (3)–(5) [38]. The discretized form of the integration can be described as

(12) $$x_{k+1}=f\left(x_{k},a_{m},\omega_{m},n_{I}\right),$$

where $n_{I}$ denotes zero-mean white Gaussian IMU measurement noise. The predicted state, x, in the prediction step is generated as

(13) $${\overset{ˇ}{x}}_{k}=f\left({\hat{x}}_{k},a_{m},\omega_{m},0\right).$$

The associated predicted state covariance matrix, $\overset{ˇ}{P}$, is propagated via

(14) $${\overset{ˇ}{P}}_{k}=\Phi_{k}{\hat{P}}_{k}\Phi_{k}^{⊤}+Q_{k},$$

where ${\hat{P}}_{k}$ is the estimated state covariance matrix from the previous iteration, and $\Phi_{k}$ and $Q_{k}$ are the system Jacobian and noise covariance matrices [39].

### 3.3. Dynamic Feature Elimination

The computed translation and rotation from the process model can also be used for calculating the corresponding fundamental matrix $F^{\prime }$ using the camera-IMU extrinsic parameters, which then be used in the dynamic point elimination method [23]. A fundamental matrix F can also be calculated using the image data. With both fundamental matrices, a distance measure between the epipolar line and the corresponding features using both matrices can be computed and denoted as $d_{k,i}^{1}$ and $d_{k,i}^{2}$, respectively, via

(15) [x,y,z]⊤=Fpf(i)ck,[x′,y′,z′]⊤=F′pf(i)ck+1

(16) dk,i1=|pf(i)ck+1⊤Fpf(i)ck|||x||2+||y||2

(17) dk,i2=|pf(i)ck⊤F′pf(i)ck+1|||x′||2+||y′||2

A threshold ρ is used to determine whether each matched feature point is static or dynamic via

(18) $$\phi_{k,i}=\left\{\begin{array}{c} 1,\;|d_{k,i}^{1}-d_{k,i}^{2}|\geq \rho \\ 0,\;|d_{k,i}^{1}-d_{k,i}^{2}|<\rho \end{array}\right.,$$

where $\phi_{k,i}$ indicates the consistency between the ith feature point of the kth frame and the fundamental matrix. $\phi_{k,i}$ is set to 0 if the feature point is consistent and 1 if otherwise.

Additionally, there can be a case when IMU data is invalid for the elimination method. To combat this issue, we use an additional threshold μ to keep track of match count.

(19) $$\psi_{k}=\left\{\begin{array}{c} 0,\;\sum_{i=1}^{m}\phi_{k,i}<\mu \\ 1,\;\sum_{i=1}^{m}\phi_{k,i}\geq \mu \end{array}\right..$$

The dynamic point elimination will only use the IMU data if and only if the data are valid (i.e., $\psi_{k}=0$) and trustworthy. Alternatively, if the confidence in the IMU-based dynamic point elimination is insufficient, the system switches to a RANSAC-based outlier rejection scheme.

### 3.4. Measurement Update

After deriving the camera measurement functions from the provided measurement model in Equation (9) and wheel odometry from the wheel-slip compensation, the estimation of the robot’s pose x is executed through an extended Kalman filter (EKF) process. The bearing measurement model in Equation (6) is reformulated in the following manner.

(20) $$z_{m,k}=h\left({\overset{ˇ}{x}}_{k}\right)+n_{m,k}$$

where the measurement noise is assumed to be $n_{m,k}∼N\left(0,R_{m,k}\right)$. (20) is then linearized as

(21) $${\tilde{z}}_{m,k}=H_{k}{\tilde{x}}_{k}$$

where $H_{k}$ is the measurement Jacobian. Using this linearized measurement model, the EKF update equations are set as

(22) $${\hat{x}}_{k+1}={\overset{ˇ}{x}}_{k}+K_{k}{\tilde{z}}_{m,k},$$

(23) $${\hat{P}}_{k+1}={\overset{ˇ}{P}}_{k}-K_{k}H_{k}{\overset{ˇ}{P}}_{k},$$

(24) $$K_{k}={\overset{ˇ}{P}}_{k}H_{k}^{⊤}{\left(H_{k}{\overset{ˇ}{P}}_{k}H_{k}^{⊤}+R_{m,k}\right)}^{-1},$$

where $K_{k}$ is the Kalman gain.

In WO pre-integration, one common approach is to integrate the velocity kinematics directly. However, in this research, the proposed scheme uses the odometry increments estimated from the wheel slip compensation algorithm, which is described in details in the following section.

## 4. Slip Compensation

**Figure 2:**
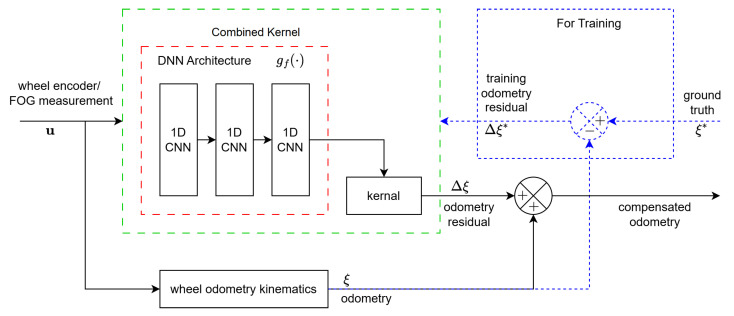
Slip compensation scheme.

Instead of directly integrating the twist of a robot for the fusion with MSCKF state estimator, the proposed algorithm utilizes slip-compensated estimation to obtain a more accurate robot pose instead. In this section, we describe the process to estimate the wheel slippage using GPR as well as the methodology used to construct the kernel to encapsulate a deep architecture. Our slip compensation design, as seen in Figure 2, involves a learning-based estimation of the (error) residuals between ground truth and physical WO model considering a planar motion differential drive robot. The goal of this scheme is to obtain more accurate robot poses based on a sequence of given velocity inputs from the encoders and the fiber optic gyroscope (FOG).

**Figure 3:**
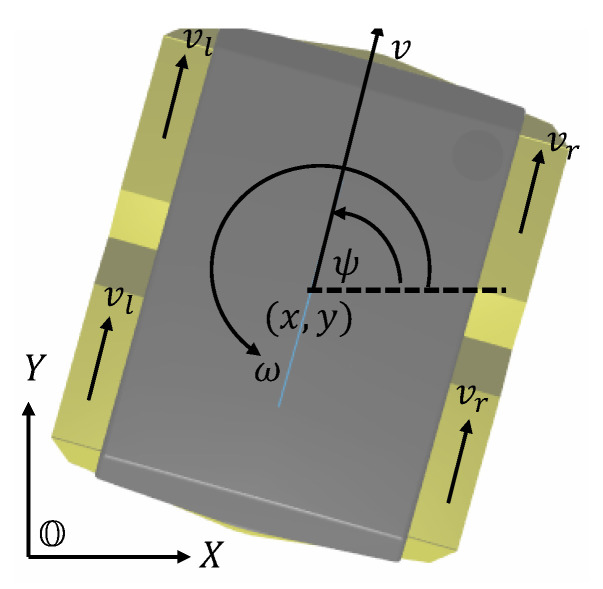
Robot kinematic schematic.

Instead of a full 6-DOF robot pose, a simplified pose (see Figure 3) is represented by the state vector

(25) $$\xi_{k}=\left[\begin{array}{c} \xi_{1k}\\ \xi_{2k}\\ \xi_{3k} \end{array}\right]={\left[\begin{array}{ccc} x_{k} & y_{k} & \psi_{k} \end{array}\right]}^{⊤},$$

where $\left(x_{k},y_{k}\right)$ denotes the position of the robot, and $\psi_{k}$ denotes yaw angle of the robot at training sample *k* for the residual estimator design in the sequel, respectively.

The velocity of both left and right wheels, $v_{l}$ and $v_{r}$, and the yaw angle that is converted from the pre-integrated IMU data, ψ, are used to obtain the robot’s differential odometry, u, based on the forward kinematics of the differential drive mobile robot,

(26) $$u_{k}=\left[\begin{array}{c} v_{k}\\ \Delta \psi_{k} \end{array}\right]=\left[\begin{array}{c} \frac{1}{2}\left(v_{l_{k}}+v_{r_{k}}\right)\\ \psi_{k+1}-\psi_{k} \end{array}\right],$$

where $v_{k}$ and $\Delta \psi_{k}$ are the predicted linear and angular velocity of the robot based on the encoders and the FOG. The obtained differential odometry can be transformed and integrated into the corresponding positional state increment Δξ using the discrete-time dynamic system model [40]

(27) $$\begin{array}{ccc} \Delta \xi_{k} & = & \xi_{k+1}-\xi_{k}={\bar{u}}_{k}+w_{k},\\ {\bar{u}}_{k} & = & \left[\begin{array}{c} {\bar{u}}_{1k}\\ {\bar{u}}_{2k}\\ {\bar{u}}_{3k} \end{array}\right]=f_{o}\left(u_{k}\right)=\left[\begin{array}{c} v_{k}\cos\left(\Delta \psi_{k}\right)\Delta_{k}\\ v_{k}\sin\left(\Delta \psi_{k}\right)\Delta_{k}\\ \Delta \psi_{k} \end{array}\right],\;w_{k}=\left[\begin{array}{c} w_{1k}\\ w_{2k}\\ w_{3k} \end{array}\right], \end{array}$$

where $w_{k}$ denotes the Gaussian process noise such that $w_{ik}∼N\left(0,\sigma_{w_{ik}}^{2}\right)$ and Δk denotes the sampling time.

In training of this learning-based estimator design utilizing GPR, which is formulated for predictive correction of the odometry increments $\Delta \xi_{k}$ governed by (27), consider a sequence of inputs (from training data sequences) collected in the individual input vector ${\bar{U}}_{i}={\left[u_{i_{k}},\cdots ,u_{i_{kn}}\right]}^{⊤}\in R^{n}$ and the corresponding sequence of state increments collected in the vector $\Delta X_{i}={\left[\Delta \xi_{i_{k}},\cdots ,\Delta \xi_{i_{k_{n}}}\right]}^{⊤}\in R^{n}$.

**Figure 4:**
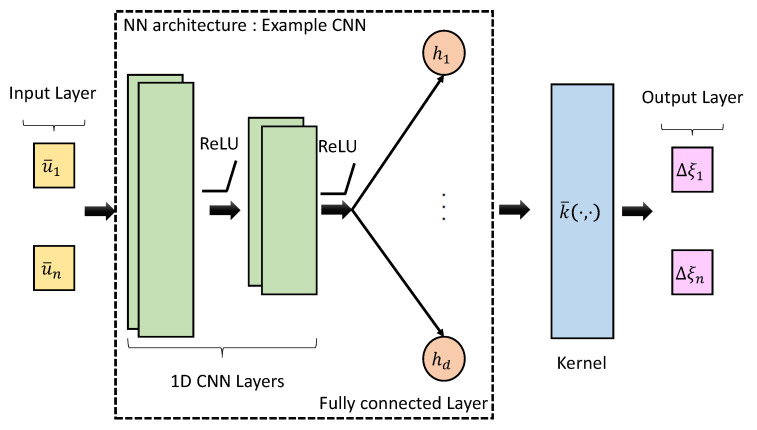
Deep kernel architecture using CNN.

For predictions $\Delta X_{i}^{*}={\left[\Delta \xi_{i_{k}}^{*},\cdots ,\Delta \xi_{i_{km}}^{*}\right]}^{⊤}$ on new data ${\bar{U}}_{i}^{*}\in R^{m}$ (test sequences), the predictive inference is given by

(28) $$p\left(\Delta X_{i}^{*}|{\bar{U}}_{i},\Delta X_{i},{\bar{U}}_{i}^{*}\right)∼N\left(\Delta {\bar{X}}_{i}^{*},\mathrm{cov}\left(\Delta X_{i}^{*}\right)\right),$$

where

(29) $$\Delta {\bar{X}}_{i}^{*}=K\left({\bar{U}}_{i}^{*},{\bar{U}}_{i}\right){\left[K\left({\bar{U}}_{i},{\bar{U}}_{i}\right)+\sigma_{w_{i}}^{2}I\right]}^{-1}\Delta X_{i}$$

is the mean prediction, and

(30) $$\begin{array}{cc} \mathrm{cov}(\Delta {\bar{X}}_{i}^{*}) & =K({\bar{U}}^{*},{\bar{U}}^{*})-\\ & K\left({\bar{U}}^{*},\bar{U}\right){\left[K\left(\bar{U},\bar{U}\right)+\sigma_{w_{i}}^{2}I\right]}^{-1}K\left(\bar{U},{\bar{U}}^{*}\right) \end{array}$$

is the prediction covariance; $K\left({\bar{U}}^{*},{\bar{U}}^{*}\right)\in R^{m\times m}$, $K\left({\bar{U}}^{*},\bar{U}\right)\in R^{m\times n}$, $K\left(\bar{U},{\bar{U}}^{*}\right)\in R^{n\times m}$, and $K\left(\bar{U},\bar{U}\right)\in R^{n\times n}$ are the kernel matrices where the element (i,j) of $k_{ij}=k\left(u_{i},u_{j}\right)$. k(·,·) is the kernel function, which consists of the Matern kernel [41] as the base kernel $\bar{k}$ combined with the non-linear mapping given by the neural network (NN) architecture $g_{f}\left(\cdot \right)$. As shown in Figure 4 the kernel function can be transformed into a deep kernel architecture. Hence, the inputs are transformed as [42]

(31) $$k\left({\bar{u}}_{i},{\bar{u}}_{j}|\Theta \right)=\bar{k}\left(g_{f}\left({\bar{u}}_{i},w\right),g_{f}\left({\bar{u}}_{j},w\right)\right),$$

with the Matern kernel function $\bar{k}\left(\cdot ,\cdot \right)$ given as

(32) $$\bar{k}\left({\bar{u}}_{i},{\bar{u}}_{j}|\Theta \right)=\sigma_{k}^{2}\left(1+\frac{\sqrt{5}\left|{\bar{u}}_{i}-{\bar{u}}_{j}\right|}{l}+\frac{\sqrt{5}{\left|{\bar{u}}_{i}-{\bar{u}}_{j}\right|}^{2}}{3l^{2}}\right)Exp\left(-\frac{\sqrt{5}\left|{\bar{u}}_{i}-{\bar{u}}_{j}\right|}{l}\right)$$

where $\sigma_{k}$ is the magnitude parameter and l>0 is the scale parameter, which are hyperparameters of the kernel. The input to the kernel function is warped by the non-linear mapping $g_{f}\left(\bar{u},w\right)$, which includes ReLU activation functions where the NN architecture is parameterized by the weights w. We construct two deep kernel architectures utilizing convolutional neural networks (CNNs) and recurrent neural networks (RNNs). The CNN-based architecture follows two layers of 1D CNN layers followed by a fully connected layer. The LSTM-based architecture follows two layers of LSTM followed by a fully connected layer.

In the training process, the hyperparameters γ={Θ and w}, which include the weights w of the NN network and $\Theta =\{\sigma_{k},l\}$ the parameters of the base kernel $\bar{k}\left(\cdot ,\cdot \right)$, are learned and optimized. The magnitude parameter $\sigma_{k}$ dictates the vertical span of the kernel function in (32), and the scale parameter *l* dictates the rate at which the correlation between two points decreases with increasing distance. Table 1 provides the pre-defined learning parameters used. These parameters are initially set by trial and error and this library [43] was used to develop the training function that optimized the hyperparameters. As shown in Figure 4, we use the ReLU activation function for CNN-based deep kernel architecture. In addition, sigmoid and tanh activation functions are used for RNN-based deep kernel architecture. The computational bottleneck of the system for inference is the computation of ${\left[K\left(\bar{U},\bar{U}\right)+\sigma_{w_{i}}^{2}I\right]}^{-1}$. Due to the large number of inputs in the data-set, we use a sparse GPR with approximate kernel learning based on induced points. Hence, instead of having to invert an $R^{n\times n}$ matrix, we make a low rank approximation and invert an $R^{m\times m}$ matrix instead, where *m* is the number of inducing points. Using the optimized parameters, the corrected state vector increment $\Delta \xi_{i_{q}}$ for i=1,2,3, and q=1,⋯,m is obtained. Once the refined compensated set of odometry increments $\Delta \xi_{q}$ are obtained for a test sequence, they can be used by the MSCKF state estimator for updating the state.

## 5. Real-World Data Simulation

In this section, we present experimental findings utilizing the publicly available KAIST dataset [44]. Gathered in an urban environment populated by dynamic objects such as vehicles, pedestrians, and bicycles, the dataset was selected due to its incorporation of wheel encoder data, which are a feature missing in many existing visual inertial datasets. The data were collected using a robotic platform equipped with a camera, an IMU, and wheel encoders. Table 2 details the sensor specifications.

For training the GPR for slip compensation, the parameters employed were as follows: the optimizer was ‘Adam’ [45], which is an inbuilt optimizer in pytorch for stochastic optimization; the learning rate was set to 0.01; the process was trained over 100 epochs; and the sample rate was 100 Hz.

**Figure 5:**
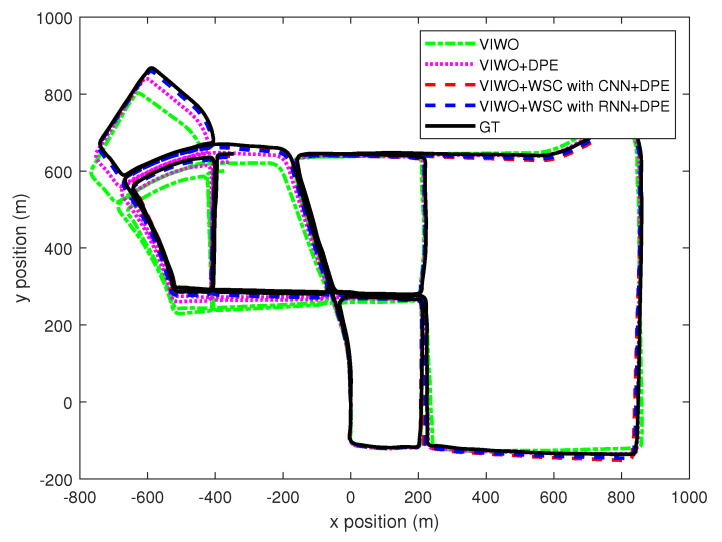
Test sequence 1 odometry trajectories comparison. VIWO: visual-inertial-wheel odometry, DPE: dynamic point elimination, WSC with CNN: wheel-slip compensation with CNN for kernel design, WSC with RNN: wheel-slip compensation with RNN for kernel design.

**Figure 6:**
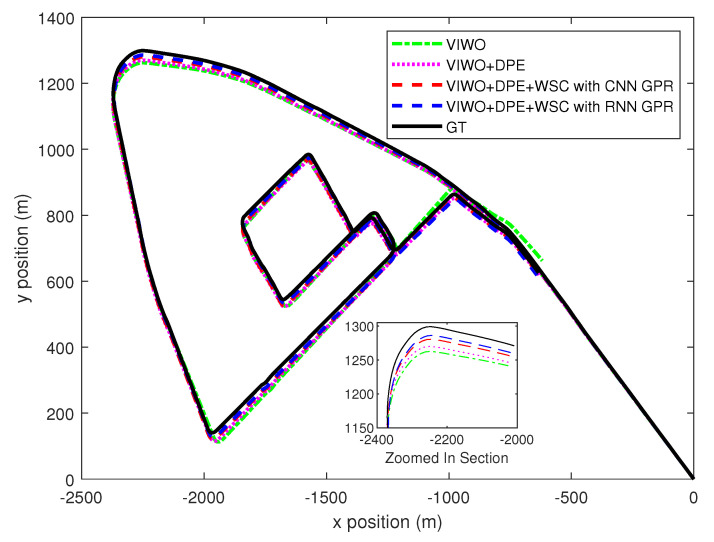
Test sequence 2 odometry trajectories comparison.

The total lengths of the two test sequences were 11.19 km and 7.6 km, respectively. The paths for both test sequences are shown in Figure 5 and Figure 6, respectively. To quantify the enhancement provided by our proposed method, we measured the mean absolute trajectory error (MATE) and mean absolute rotation error (MRTE) against the ground truth trajectory. We examined three variations of multi-state constraint Kalman filters (MSCKFs): a standard MSCKF VIWO, a MSCKF VIWO with dynamic feature point elimination, and a MSCKF VIWO incorporating both dynamic feature point elimination and wheel slip compensation. Table 3 demonstrates the superior alignment of the ground truth with the third approach, which combined slip compensation and dynamic point removal.

**Figure 7:**
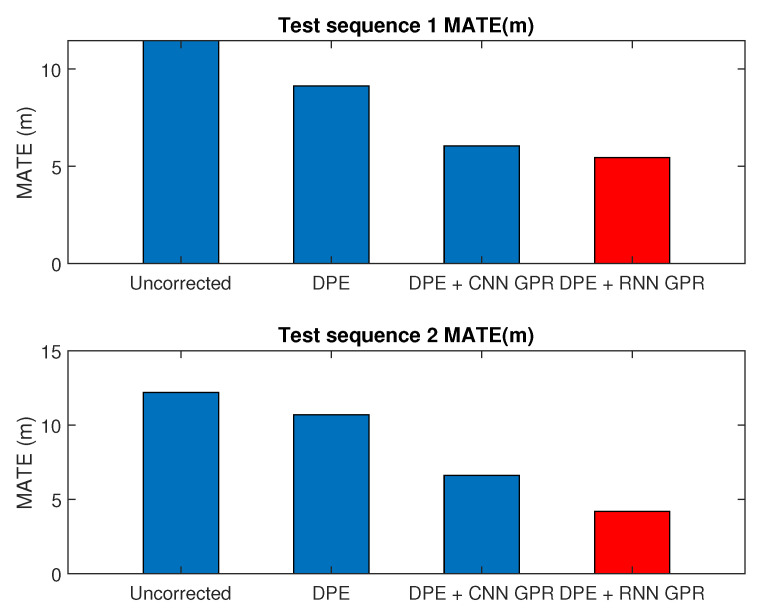
MATE error for test sequences.

In the case of VIWO without the incorporation of slip compensation and dynamic feature point elimination, the MATE/MRTE recorded was 10.825 m. However, this error reduced to 6.121 m with the integration of these features, indicating a substantial decrease in both MATE and MRTE, which are shown in Figure 7.

## 6. Indoor Experimentation

**Figure 8:**
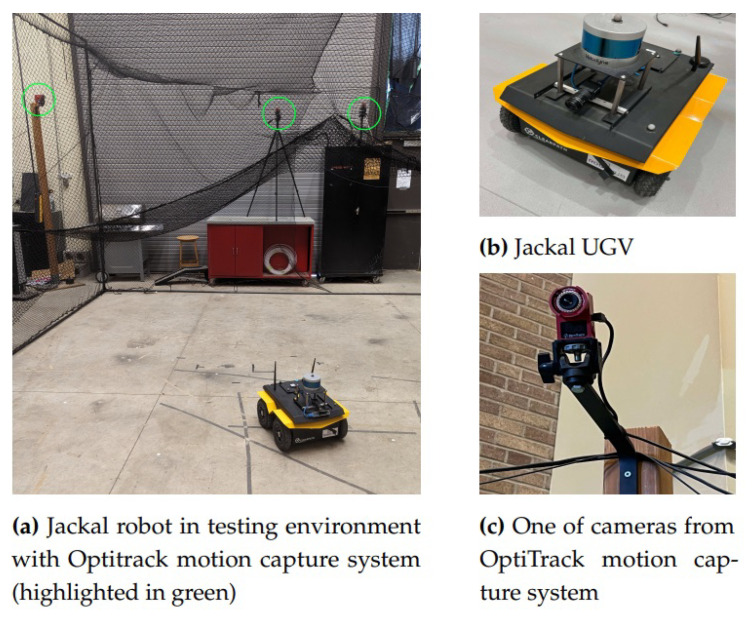
Experimental setup.

We validate our proposed methodology by conducting experiments in an indoor setup. We implement and test the scheme using Clearpath Robotics’ Jackal unmanned ground vehicle (UGV) (Clearpath Robotics, Kitchener, ON, Canada), as shown in Figure 8, equipped with readily available sensors such as wheel encoders and an inertial measurement unit (IMU). We also attach an Intel RealSense T265 tracking camera (Intel Corporation, Santa Clara, CA, USA) to obtain visual data. The Jackal UGV is a skid-steer differential-drive robot that consists of four wheels, where each pair of wheels on the left and right sides is actuated by a single motor. Hence, for modeling, it can be represented as a standard two-wheel differential-drive system. The sensor specifications and associated measurements can be found in Table 4. The data from the Jackal UGV are obtained via Robot Operating System (ROS2 Humble) [46]. To validate the experimental results, we compare them with data obtained from the OptiTrack motion capture system (NaturalPoint, Inc., Corvallis, OR, USA), which serves as the ground truth reference.

**Figure 9:**
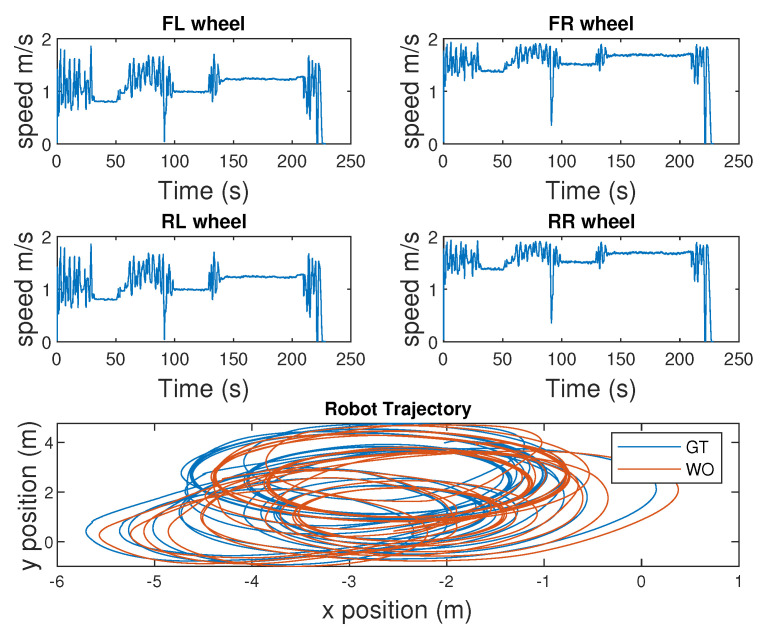
Comparison of wheel speeds of the robot.

Figure 9 illustrates the difference in wheel speeds when turning and traversing in an arc and the associated slippage.

**Figure 10:**
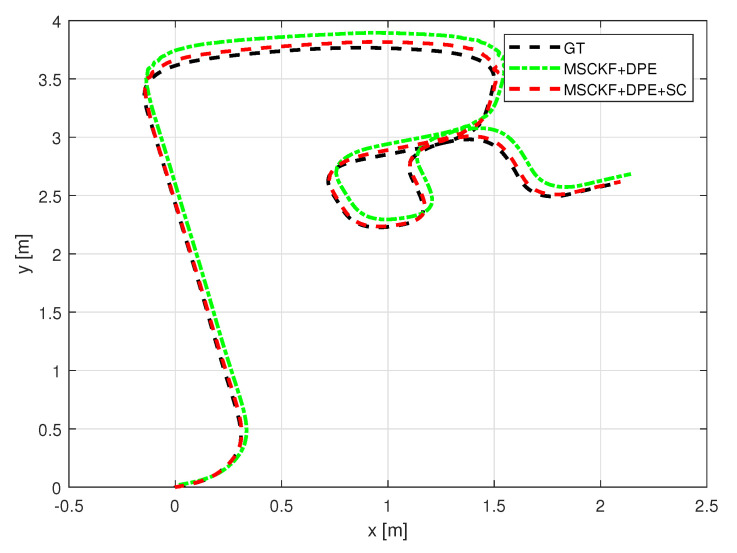
Experiment sequence 1 odometry trajectory comparison.

**Figure 11:**
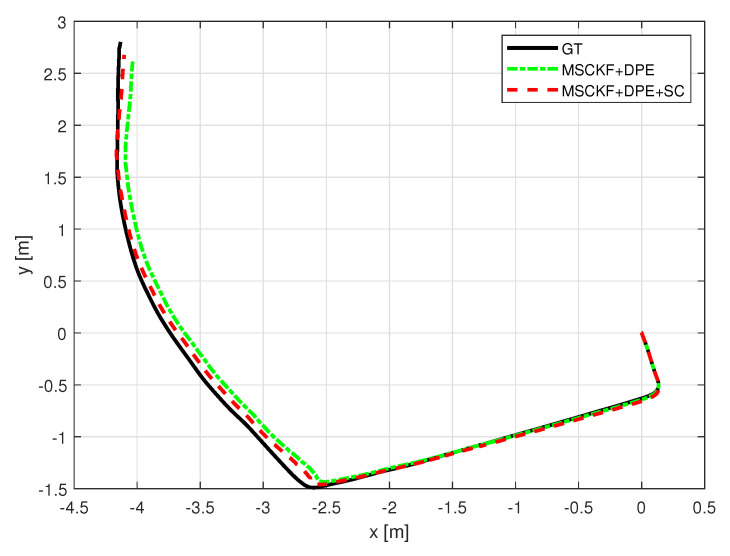
Experiment sequence 2 odometry trajectory comparison.

**Figure 12:**
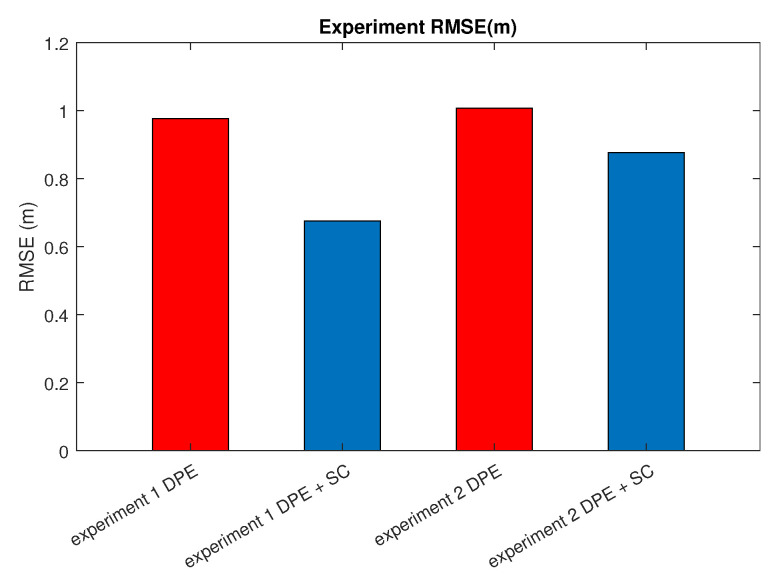
RMSE for the experimental trajectories.

With the indoor set-up, we tested the proposed algorithm under the same metrics as in Section 5. Figure 10 and Figure 11 depict the robot trajectories for the cases, which include slip compensation plotted against the ground truth trajectory obtained with the optitrack motion capture system. As seen in Figure 10 and Figure 11, which displays the barplots for RMSE(m), the trajectory obtained via augmented slip compensation and dynamic feature point elimination had the lowest RMSE, as shown in Figure 12. In summary, our methodology was able to increase the accuracy of localization for the outdoor real-world data, as well as the indoor experiments conducted using a UGV.

**Figure 13:**
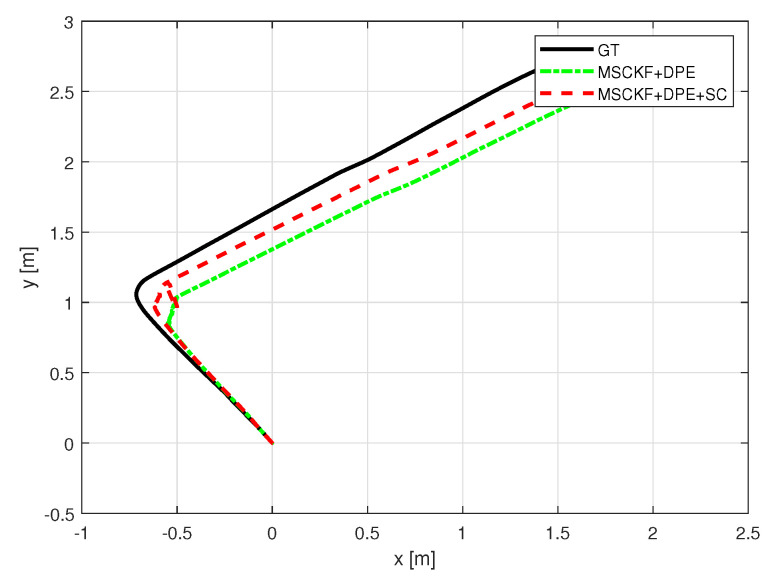
Experiment sequence 3 odometry trajectories comparison in gravel/sand-based terrain.

To further test our methodology, we used gravel and sand on the test floor to determine the effectiveness of the proposed system. Figure 13 shows the robot trajectories for when the navigating surface is layered with gravel and sand. Our proposed scheme was able to improve the localization accuracy through aiding in slippage compensation.

## 7. Conclusions

In this study, we introduced a data-driven Gaussian process regression (GPR) approach for wheel slip compensation, paired with a dynamic feature elimination mechanism to enhance visual-inertial-wheel odometry (VIWO)-aided navigation systems. The proposed slip compensation scheme incorporates advanced deep kernel designs and long short-term memory (LSTM) network layers, which are adept at capturing spatiotemporal variations in odometry errors. A core component of the scheme is the feature point confidence estimator, which plays a pivotal role in mitigating the adverse effects of dynamic feature points on visual measurements. By refining these measurements, the system achieves improved pose estimation accuracy. Furthermore, inertial measurements are corrected and fused with wheel encoder data through the multi-state constraint Kalman filter (MSCKF), ensuring robust state estimation. The effectiveness of the approach was validated through extensive real-world data simulations and controlled indoor experiments, demonstrating its superiority in reducing errors and enhancing localization accuracy under various operational conditions.

Extensive real-world outdoor experiments using the KAIST dataset and controlled indoor tests with a Clearpath Jackal UGV validated the effectiveness of our approach. In outdoor experiments, our method reduced the mean absolute trajectory error (MATE) from 11.45 m to 5.45 m and the mean absolute rotation error (MRTE) from 2.526° to 1.079°. Similarly, in indoor trials using OptiTrack as ground truth, the proposed scheme significantly reduced RMSE, particularly on challenging terrains such as gravel and sand, demonstrating its robustness in varying conditions.

Looking ahead, the proposed framework sets the foundation for several promising research directions. One key area of improvement is enhancing the neural network architecture used for slip compensation. Future work will explore more advanced deep learning models, such as transformer-based architectures and convolutional-recurrent hybrid networks, to improve the system’s ability to capture complex temporal dependencies in slip dynamics. Additionally, integrating unsupervised and self-supervised learning techniques into the slip compensation process could enable the system to adapt autonomously to new environments and conditions, further improving its robustness without the need for extensive labeled data. Furthermore, refining the system architecture to incorporate terrain-aware modeling using deep learning techniques could allow real-time adjustments based on surface characteristics such as friction, inclination, and deformability. This would improve adaptability in dynamic and challenging environments, making the system less sensitive to shifts in slip dynamics. Such advancements would broaden the applicability of the system to diverse scenarios, including off-road navigation, disaster response, and industrial automation. The insights gained from this research highlight the potential of combining advanced neural-network-based slip compensation with multi-sensor fusion, paving the way for more reliable and efficient autonomous navigation systems.

## Figures and Tables

**Table 1:** Parameters for training GP.

Training Parameter	Value
Optimizer	Adam
Learning rate	0.01
No. of epochs	100
sample rate	100 Hz
Kernel length parameter	1

**Table 2:** Sensor specifications for KAIST dataset.

Sensor	Manufacturer	Model	Description	Freq. (Hz)
Camera	Pointgray	Flea3	1600 × 1200 color, 59 FPS	10
IMU	Xsens	MTi-300	Enhanced AHRS Gyro	100
wheel encoder	RLS	LM13	Magnetic rotary encoder	100

**Table 3:** MATE/MRTE KAIST dataset.

Method	MATE [m]	MRTE [Degree]
VIWO uncorrected	11.45	2.526
VIWO with DPE	9.13	1.912
VIWO with DPE and CNN GPR	6.04	1.279
VIWO with DPE and RNN GPR	5.45	1.079

**Table 4:** Jackal Sensor specifications.

Sensor	Description	Frequency
Camera	Intel realsense T265	10 fps
IMU		400 Hz
Wheel encoders	78,000 pulses/m quadrature	100 Hz

## Data Availability

The data supporting the findings of this study are openly available at https://github.com/UW-CAMS-Lab/VIWO-SLIP accessed on 23 January 2025.

## Appendix A

**Table A1:** Comparative analysis of the proposed approach’s key features with respect to some related VIWO/VINS designs in the literature.

Category	Proposed Approach	VINS with WO [1,6,24]	Filter-Based VIWO [5,25,35]	VIWO with Slip Detection [20]
**Objective**	Compensate for wheel slippage and improve VIWO accuracy.	Refine inertial measurements and improve the accuracy of VIWO.	Fuse WO and VINS for improved localization.	Detect and mitigate slip in real time.
**Methodology**	GPR and LSTM to model and correct slip errors; integrates a feature confidence estimator.	Neural gyroscope calibration to reduce drift in localization.	Partial invariant EKF (PIEKF) to optimally fuse WO and VINS.	Threshold-based slip detection and rejection.
**Key Innovation and Limitations**	Integrates adaptive slip correction with dynamic feature elimination for robust VIWO.	Uses deep learning for enhanced gyroscope drift compensation. Affected by wheel slip and inherent IMU drifts, leading to localization errors. Ref. [24] has focused on reducing the gyroscope drift.	Leverages kinematic constraints to refine odometry accuracy but assumes minimal slip, causing errors in dynamic conditions.	Ensures real-time slip detection but does not model slip dynamics, leading to accumulated drift over time.

**Table A2:** Comparative feature summary of the proposed approach with respect to some recently developed deep-learning-based VO algorithms in the literature.

Feature	Proposed	GNSS-Free UAV Navigation [28]	VO Using RCNNs [29]	VO Using CNN and Bi-LSTM [30]
**Objective**	Uses GPR and LSTM to compensate for wheel slippage, enhancing VO accuracy.	LSTM-based GNSS-free UAV navigation using optical odometry and radar.	Monocular VO with deep RCNNs estimating poses from raw RGB images.	Monocular VO with CNN and Bi-LSTM for 6-DoF pose estimation.
**Methodology**	GPR-LSTM fusion to correct slippage-induced errors in VO.	LSTM for drift correction and radar for scale estimation.	CNN for feature extraction, RNN for sequential motion modeling.	Hybrid CNN for features, Bi-LSTM for sequence learning.
**Key Innovation and Limitations**	Combines GPR and LSTM for adaptive slippage compensation, improving localization.	Uses LSTM for velocity correction and radar for scale resolution.	End-to-end learning without explicit VO components.	Bi-LSTM utilizes past and future frames for improved estimation.
**Main Contributions**	Enhances wheel odometry with ML and sensor fusion.	Reliable UAV navigation in GNSS-denied areas.	VO without feature engineering or scale priors.	End-to-end VO without geometric constraints.
**Technological Focus**	Ground navigation with: (1) Slippage compensation, (2) Robust localization, (3) Improved odometry.	UAV navigation, GNSS signal loss mitigation.	Learning-based feature extraction and motion estimation.	Enhancing feature extraction and motion estimation.
**Validation**	Tested on KAIST dataset, showing robust performance in dynamic terrains.	Flight-tested, reducing velocity errors in GNSS outages.	Validated on KITTI dataset, competitive with traditional VO.	Tested on KITTI, ETH-asl-cla, suited for structured driving.
**Advantages**	Accurate slippage compensation, improving localization in uneven terrains.	Reliable UAV navigation under GNSS loss.	Adaptable VO without manual tuning.	No manual feature extraction, robust across environments.
